# Supplementary material for: Botulinum Toxin Type A for Pediatric Torticollis: A Review of Clinical Research
Source: Toxins (Basel). 2025 Nov 1;17(11):543. doi: 10.3390/toxins17110543 (PMC12656473; doi:10.3390/toxins17110543)
Supplement: Supplementary file 1 [file toxins-17-00543-s001.zip › toxins-3877995-supplementary/Table S2. Search strategies and search terms used in each database.pdf]

Table S2. Search strategies and search terms used in each database

Medline via PubMed (15.7.2025.)

	Searches
#1	"Botulinum Toxins, Type A"[MH] OR "Botulinum toxin type A"[TIAB]
#2	"Congenital torticollis"[MH] OR "Pediatric torticollis"[TIAB] OR "Congenital muscular torticollis"[TIAB]
#3	"Clinical trial"[PT] OR "Randomized controlled trial"[PT] OR "Controlled clinical trial"[PT] OR "Case reports"[PT]
**#4**	**#1 AND #2 AND #3**

Embase via Elsevier (15.7.2025.)

	Searches
#1	'botulinum toxin a'/exp OR ' botulinum toxin a':ab,ti
#2	'pediatric torticollis'/exp OR 'pediatric torticollis':ab,ti OR 'congenital muscular torticollis'/exp OR 'congenital muscular torticollis':ab,ti
#3	'clinical trial'/exp OR 'clinical trial':ab,ti OR 'randomized controlled trial'/exp OR 'randomized controlled trial':ab,ti OR 'controlled clinical trial'/exp OR 'controlled clinical trial':ab,ti OR 'case report'/exp OR 'case report':ab,ti
**#4**	**#1 AND #2 AND #3**

CENTRAL (15.7.2025.)

	Searches
#1	MeSH descriptor: [Botulinum Toxins, Type A] explode all trees
#2	(botulinum toxin type a):ti,ab,kw
#3	#1 OR #2
#4	((pediatric torticollis) OR (congenital muscular torticollis)):ti,ab,kw
#5	MeSH descriptor: [Clinical Trial] explode all trees
#6	MeSH descriptor: [Randomized Controlled Trial] explode all trees
#7	MeSH descriptor: [Controlled Clinical Trial] explode all trees
#8	MeSH descriptor: [Case Reports] explode all trees
#9	((clinical trial) OR (randomized controlled trial) OR (controlled clinical trial) OR (case report)):ti,ab,kw
#10	#5 OR #6 OR #7 OR #8 OR #9
**#11**	**#3 AND #4 AND #10**

CINAHL (EBSCOhost) (13.6.2025.)

	Searches
#1	TX "botulinum toxin type a"
#2	TX "pediatric torticollis" OR "congenital muscular torticollis"
#3	MH "Clinical Trials"
#4	MH "Randomized Controlled Trials"
#5	MH "Nonrandomized Trials"
#6	MH "Single-Blind Studies"

#7	TX "clinical trial" OR "randomized controlled trial" OR "controlled clinical trial" OR "case report"
#8	#3 OR #4 OR #5 OR #6 OR #7
**#9**	**#1 AND #2 AND #8**

Korean medical databases - ScienceON, Korean traditional knowledge portal, Korea Citation Index,

Research Information Sharing Service, OASIS, and Korean Medical database (13.6.2025.)

	Searches
#1	"botulinum toxi atric torticollis" OR "congenit n type a" OR "보툴리눔 톡신 A"
#2	"pediatric torticollis" OR botulinum toxin type a "congenital muscular torticollis" OR "소아 사경" OR "선천선 근육성 사경"
#3	"clinical trial" OR "randomized controlled trial" OR "controlled clinical trial" OR "case report" OR "임상시험" OR "무작위 대조군 임상연구" OR "임상연구" OR "증례 보고"
#4	#1 AND #2 AND #3

Korean medical databases – CNKI and Wanfang (13.6.2025.)

	Searches
#1	"botulinum toxin type a" OR "肉毒杆菌毒素 A型"
#2	"pediatric torticollis" OR "congenital muscular torticollis" OR "儿童斜颈" OR "先天性肌性斜颈"
#3	"clinical trial" OR "randomized controlled trial" OR "controlled clinical trial" OR "case report" OR "临床研究" OR "随机对照试验" OR "病例报告"
#4	#1 AND #2 AND #3
